# Integrative Transcriptomics and Machine Learning Identify Macrophage-Associated Biomarkers in Hypertrophic Cardiomyopathy

**DOI:** 10.3390/ijms27115102

**Published:** 2026-06-04

**Authors:** Jianzhi Zhao, Ximiao Su, Jiali Wu, Yanan Qin, Chengyu Song, Yanli Li, Chang Liu, Ran Li, Qiushi Wang, Chen Liang

**Affiliations:** Departments of Cardiology, Central Laboratory, The First Affiliated Hospital of Harbin Medical University, Harbin 150010, China; 19254500534@163.com (J.Z.); suximiao51@163.com (X.S.); 2023020729@hrbmu.edu.cn (J.W.); 18947164307@163.com (Y.Q.); 2024020762@hrbmu.edu.cn (C.S.); 2024020763@hrbmu.edu.cn (Y.L.); 2025021012@hrbmu.edu.cn (C.L.); 2025021011@hrbmu.edu.cn (R.L.)

**Keywords:** hypertrophic cardiomyopathy, single-cell sequencing, machine learning, macrophage, immune infiltration

## Abstract

Hypertrophic cardiomyopathy (HCM) is a common genetic heart disease, with macrophages playing a critical role in its pathological remodeling. Our study aims to investigate the molecular basis of HCM by analyzing macrophage-related gene expression at the single-cell level. Utilizing published scRNA-seq datasets (GSE181764 and GSE161921), we identified macrophages as the key cell cluster most associated with HCM. Integration with bulk RNA-seq data (GSE249925) and differential expression analysis revealed three hub genes: *ASPN* (asporin), *F13A1* (Coagulation Factor XIII A Chain), and *SORBS2* (Sorbin and SH3 domain-containing protein 2). Immune infiltration analysis showed significant decreases in multiple immune cell subsets in HCM patients, including neutrophil and macrophages. Intercellular communication analysis revealed an approximately 50% reduction in total interactions in HCM, accompanied by markedly weakened macrophage signaling reception and loss of regulatory pathways. Single-cell validation confirmed that *F13A1* expression was predominantly restricted to macrophage clusters and significantly downregulated in HCM macrophages, demonstrating strong macrophage specificity and diagnostic potential. Furthermore, a LASSO-based diagnostic model incorporating three genes (*IGFBP4*, *FOS*, *CTSC*) exhibited high predictive performance, with validated accuracy in both training and external validation sets. Collectively, our findings shed light on the mechanisms underlying macrophage dysfunction in HCM and offer novel insights into the cellular and molecular dynamics.

## 1. Introduction

Hypertrophic cardiomyopathy (HCM) is an autosomal dominant inherited cardiomyopathy characterized primarily by asymmetric hypertrophy of the left and/or right ventricular myocardium, with its pathophysiological basis originating from mutations in genes encoding cardiac sarcomere proteins [1]. As one of the leading causes of sudden death in young individuals, HCM not only predisposes to heart failure, atrial fibrillation, and stroke but also imposes a substantial disease and economic burden on patients’ families and the healthcare system. Despite its diverse clinical manifestations, myocardial fibrosis represents a key pathological feature of HCM, and its severity is significantly positively correlated with the risk of adverse cardiac events, particularly sudden cardiac death [2]. Current clinical diagnosis primarily relies on imaging techniques such as echocardiography and cardiac magnetic resonance imaging, the latter enabling effective assessment of myocardial fibrosis through late gadolinium enhancement [3]. However, these methods have limitations in early disease detection, accurate evaluation of disease activity, and personalized prognosis prediction, highlighting the urgent need to explore novel and reliable biomarkers to assist clinical decision-making.

Notably, the pathological progression of HCM is far from being driven solely by cardiomyocyte-autonomous hypertrophy and dysfunction. Accumulating evidence indicates that dysregulation of the cardiac immune microenvironment, particularly the infiltration and activation of innate immune cells, plays a critical role in myocardial inflammation, fibrosis, and adverse remodeling [4]. Among these, macrophages, as a core population of resident and recruited immune cells in the heart, exert dual functions in maintaining tissue homeostasis, clearing apoptotic cells (i.e., efferocytosis), and coordinating repair and fibrotic responses through their remarkable phenotypic plasticity [5]. Previous studies have suggested that the phenotype of macrophages in the hearts of HCM patients may shift, for instance toward a pro-inflammatory phenotype, thereby influencing fibroblast activation through cytokine secretion and ultimately exacerbating myocardial fibrosis [6]. Moreover, deficiency of the macrophage-specific molecule NLRC5 has been shown to aggravate pressure overload-induced pathological cardiac remodeling by intensifying inflammatory responses [7]. Nevertheless, most of these findings are based on specific models or limited samples, and a systematic characterization of the overall immune landscape of the HCM heart at single-cell resolution—particularly the detailed alterations of macrophage subpopulations, their dynamic communication networks with other cardiac cells (e.g., cardiomyocytes and fibroblasts), and the precise functional implications of these changes in HCM pathogenesis—remains lacking.

To address these knowledge gaps, this study adopted an innovative analytical strategy integrating transcriptomic data. Recent studies have begun to apply single-cell or single-nucleus sequencing to explore the cellular landscape of HCM; for example, in pediatric end-stage HCM or mouse models carrying specific gene mutations, phenomena such as cardiomyocyte stress, fibroblast activation, and diminution of tissue-resident macrophages have been observed [8,9]. However, these studies either focus on specific patient populations or are confined to single models, and none have systematically integrated scRNA-seq and bulk RNA-seq data from different cohorts in public repositories to comprehensively delineate the cellular composition of the adult HCM heart and precisely identify disease-relevant core genes of macrophages. Furthermore, although machine learning has shown promise in HCM diagnosis—for instance, in constructing models based on electrocardiographic, radiomic, or metabolomic data [10,11]—studies that directly develop diagnostic models using gene signatures with well-defined cellular origins derived from the HCM cardiac microenvironment remain rare.

Therefore, the central aim of this study is to systematically reveal the remodeling of the cardiac immune microenvironment, particularly the macrophage-mediated intercellular communication network, in HCM by integrating single-cell and bulk transcriptomic data from human heart tissues. Specifically, we will first construct a high-resolution single-cell atlas of HCM and non-failing control hearts, quantifying and comparing the proportional changes to major cell types, especially macrophages. Second, through cross-analysis, we will identify macrophage-associated genes that are specifically altered in HCM. Third, using cell–cell communication analysis tools, we will deeply investigate the global alterations of the cardiac cell interaction network in HCM, with a focus on changes in the signaling sending and receiving capacities of macrophages. Finally, based on the identified key genes, we will employ advanced machine learning algorithms to construct and validate a robust and efficient diagnostic model for HCM. Different from prior bulk RNA-seq and immune deconvolution research on HCM, the present study makes three innovative advances: it combines scRNA-seq and bulk RNA-seq to screen macrophage-specific hub genes including *ASPN*, *F13A1* and *SORBS2*, which possess independent diagnostic potential; it systematically constructs intercellular communication networks and for the first time reveals defective signal reception in macrophages under HCM conditions; and it additionally develops a macrophage-originated three-gene machine learning signature consisting of *IGFBP4*, *FOS* and *CTSC*, with its diagnostic performance further verified via external datasets. Through this series of analyses, this study is expected not only to deepen the understanding of the immunopathological mechanisms underlying HCM and to reveal the potential role of macrophage dysfunction in driving disease progression, but also to provide a crucial theoretical foundation and data support for the development of novel non-invasive diagnostic biomarkers and potential immunomodulatory therapeutic targets.

## 2. Results

### 2.1. Single-Cell Landscape in Hypertrophic Cardiomyopathy Tissues

**Figure 1:**
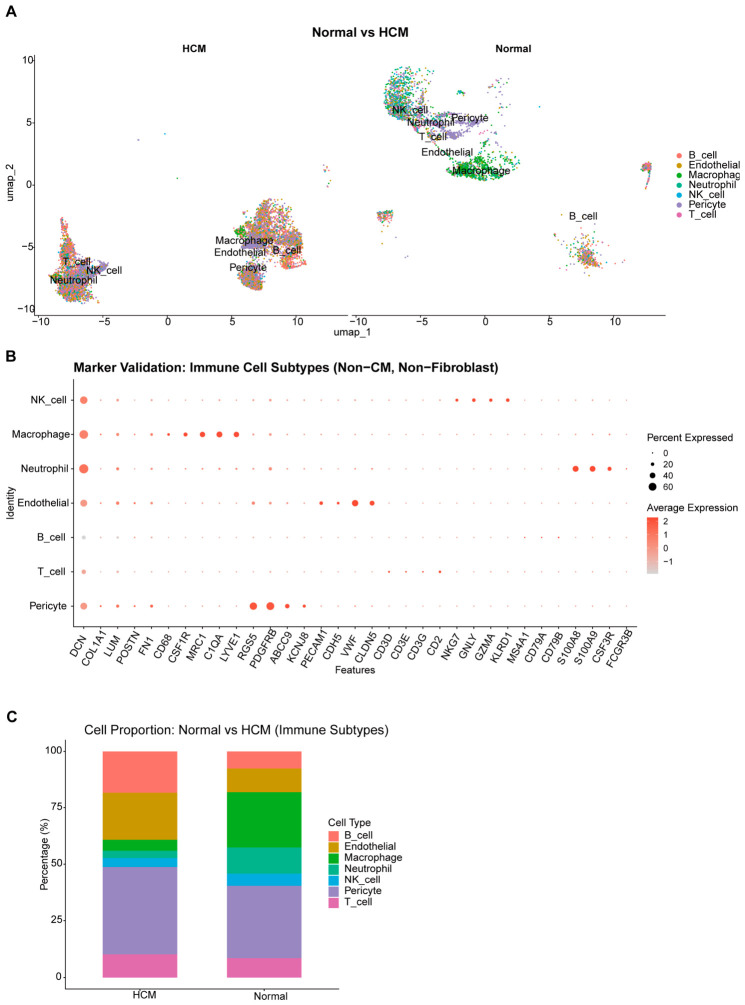
Single-cell RNA sequencing reveals major cell populations in cardiac tissues. (**A**) UMAP plot of annotated cell clusters in HCM and non-failing control groups. (**B**) Expression levels of canonical marker genes across the seven identified cell types. (**C**) Relative proportions of each cell type in HCM and non-failing control samples.

In this study, the scRNA-seq datasets GSE181764 and GSE161921 were analyzed, which comprised 7 hearts from patients with HCM and 6 non-failing control hearts. After quality control, a dataset containing 87,667 high-quality cells was obtained from single-cell samples of non-failing and HCM tissues after Harmony batch correction (Figure 1A). Notably, the scRNA-seq dataset included 4085 macrophages. Based on the expression patterns of canonical marker genes, the retained high-quality cells were classified into 5 cell types, including fibroblasts (*DCN*), macrophages (*CD68*), pericytes (*RGS5*), cardiomyocytes (*TNNT2*), and others (*PECAM1*; Figure S1A,C). Next, we visualized the proportional changes to each cell cluster between the two groups using UMAP plots (Figure S1B). Notably, a decrease in the proportion of macrophages was observed in HCM tissues relative to non-failing control samples (Figure S1D). To further delineate the dynamic changes within the immune compartment, cardiomyocytes and fibroblasts were excluded, and the remaining non-myocytes and non-fibroblasts were subjected to high-resolution annotation. This analysis identified the following subpopulations: neutrophils (*S100A8*), macrophages (*CD68*), T cells (*CD3D*), B cells (*MS4A1*), NK cells (*NKG7*), endothelial cells (*PECAM1*), and pericytes (*RGS5*) (Figure 1A,B). The results revealed a marked decrease in the proportion of macrophages in the hearts of HCM patients compared with non-failing control tissues (Figure 1C). Furthermore, the differentially expressed genes (DEGs) in cardiomyocytes, fibroblasts, macrophages, neutrophils, B cells, T cells, NK cells, pericytes, and endothelial cells were analyzed using single-cell RNA sequencing data, as presented in the Tables S1–S9. These findings indicate that dysregulation of the immune microenvironment, particularly involving macrophage abnormalities, may contribute to the pathogenesis and progression of adverse cardiac remodeling in HCM.

### 2.2. Identification of Macrophage-Related Genes

**Figure 2:**
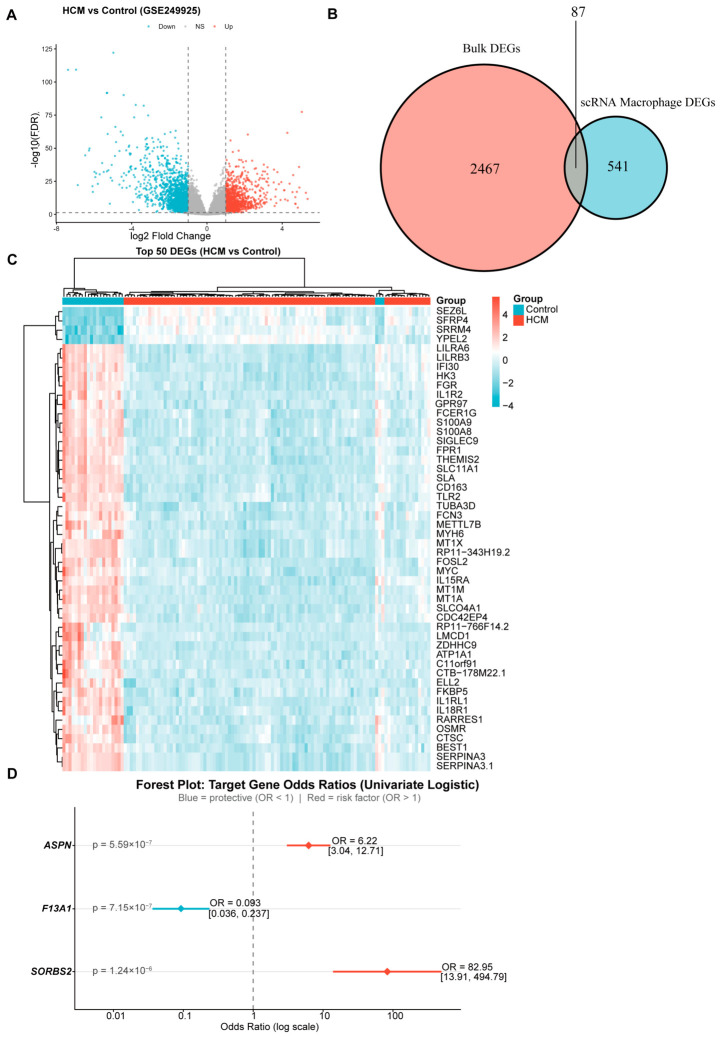
Identification of macrophage-related genes with diagnostic significance in HCM. (**A**) Volcano plot of DEGs between control and HCM groups. Red, blue, and gray nodes represent upregulated, downregulated, and non-significant genes, respectively. (**B**) Venn diagram showing the overlap between macrophage-specific genes derived from scRNA-seq data and DEGs identified from bulk RNA-seq data. (**C**) Heatmap displaying the top 50 DEGs between control and HCM groups. (**D**) Forest plots illustrating odds rations of three key genes for HCM.

Differential expression analysis was performed on bulk RNA-seq data from the GSE249925 dataset, including 97 HCM samples and 23 non-failing heart tissue samples. A total of 2554 DEGs were identified based on the criteria of |log2(FC)| > 1 and p.adj < 0.05 (Figure 2A). Out of these, 1085 genes showed an increase in expression, while 1469 genes showed a decrease in expression in HCM. The expression of Top 50 marker genes was depicted by heatmap (Figure 2C). Subsequently, by taking the intersection of macrophage-specific genes from the scRNA-seq dataset with DEGs from the bulk RNA-seq dataset, 87 macrophage-related genes were identified in HCM (Figure 2B). The 87 genes were subjected to univariate Logistic regression (86 of which were significant), and then entered multi-step stepwise multivariate regression. Eventually, 3 target genes including *ASPN* (asporin), *F13A1* (Coagulation Factor XIII A Chain), and *SORBS2* (Sorbin and SH3 domain-containing protein 2) with independent diagnostic contributions to HCM were screened out (Figure 2D).

### 2.3. Exploration of GO Term and KEGG Pathway Enrichment in DEGs

**Figure 3:**
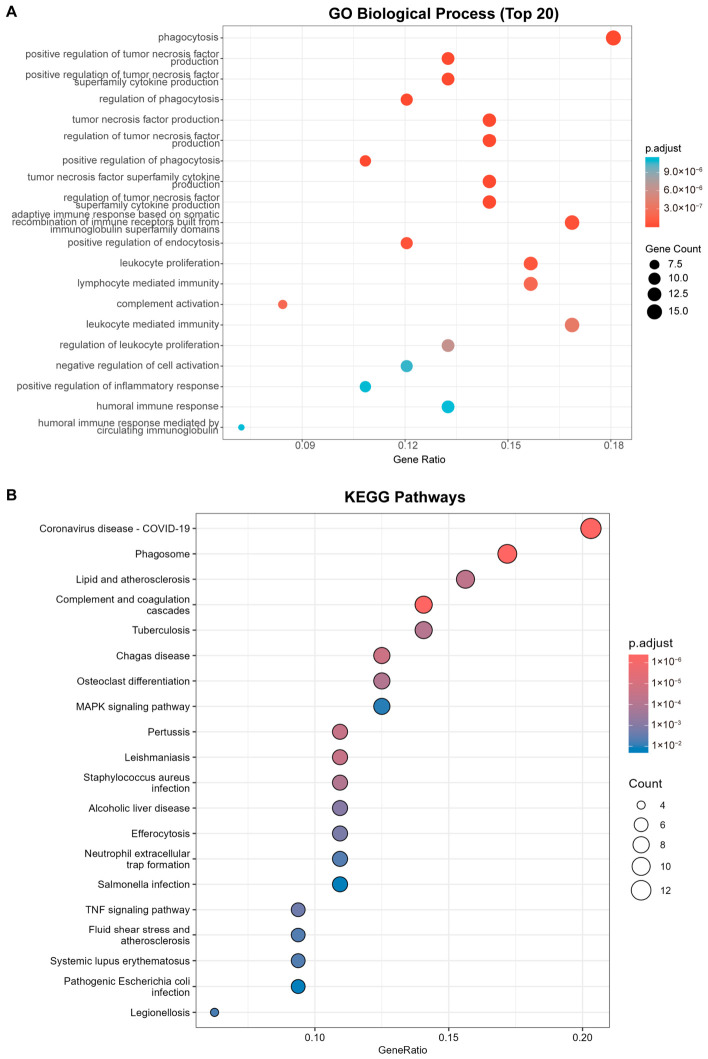
Functional enrichment analysis of DEGs in HCM. (**A**) GO enrichment biological process analysis of DEGs. (**B**) KEGG pathway enrichment analysis of DEGs.

Gene Ontology (GO) enrichment analysis was performed to explore the biological functions of DEGs shared in HCM. A total of 396 GO biological process terms were significantly associated with these DEGs. Notably, the enriched molecular functions included immunoglobulin binding, positive regulation of inflammatory response, leukocyte proliferation, and extracellular matrix (Figure 3A and Figure S2). In parallel, Kyoto Encyclopedia of Genes and Genomes (KEGG) pathway enrichment analysis was conducted to identify several key signaling pathways and visualize their interrelationships (Figure 3B). These DEGs were primarily enriched in immune cell-related pathways, including the MAPK signaling pathway, TNF signaling pathway, and complement and coagulation cascades. Collectively, the functional annotations of the DEGs were predominantly linked to macrophage activation and myocardial fibrosis, reinforcing the biological relevance of the identified target genes.

### 2.4. Immune Infiltration Analyses

**Figure 4:**
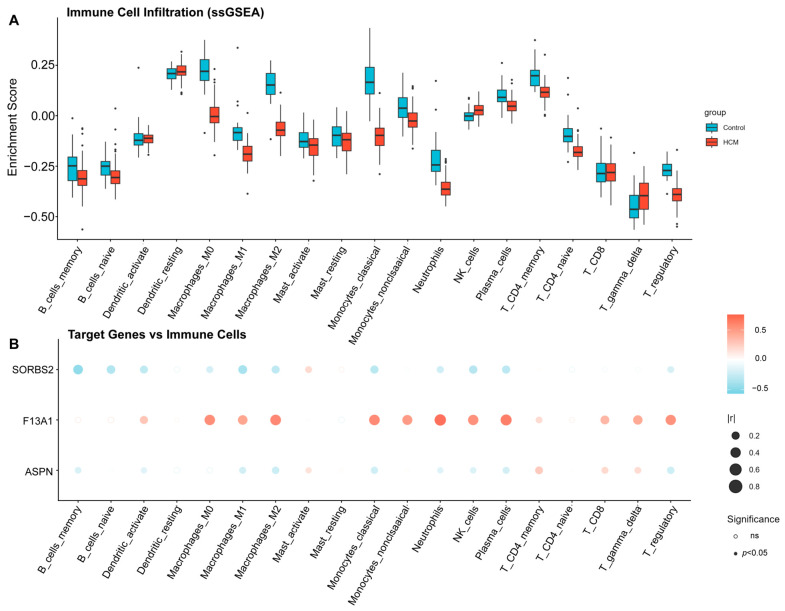
Immune infiltration analysis between HCM patients and non-failing controls. (**A**) Box plots showing ssGSEA scores of 19 immune cell types in HCM and non-failing samples. (**B**) Spearman correlation bubble plot illustrating the association between target genes (*ASPN*, *F13A1*, *SORBS2*) and the immune cells.

The single-sample gene set enrichment analysis (ssGSEA) algorithm was applied to quantify the infiltration levels of 19 immune cell types in the training set samples. Myocardial tissues from HCM patients exhibited elevated levels of gamma delta T cells and NK cells, along with reduced levels of macrophages, neutrophils, and monocytes (Figure 4A). Furthermore, correlation analysis was conducted to assess the relationship between target gene expression and immune cell infiltration. The results revealed that *F13A1* was significantly positively correlated with multiple macrophage subtypes, further supporting its functional importance in macrophages within the context of HCM (Figure 4B).

### 2.5. Analysis of Differences in CellChat Intercellular Communication

**Figure 5:**
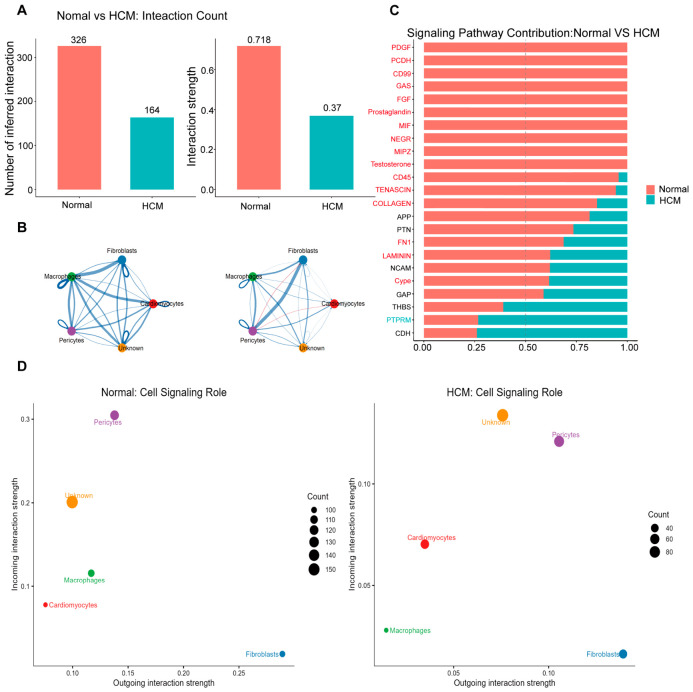
Intercellular communication analysis in HCM and non-failing cardiac tissues. (**A**) Comparison of the number (**left**) and interaction strength (**right**) of intercellular communications between HCM and non-failing heart cells. (**B**) Cell communication networks in non-failing (**left**) and HCM (**right**) samples. Line thickness indicates the intensity of intercellular interactions. (**C**) Stacked bar chart showing the relative contribution ranks of signaling pathways in non-failing and HCM samples. (**D**) Scatter plots depicting signaling emission (*x*-axis) and reception (*y*-axis) intensities for each cell type.

Using the CellChat framework, we constructed intercellular communication networks for non-failing hearts and hearts with HCM, respectively. The changes in cell communication under disease conditions were mcompared, with particular attention to signaling pathways involving macrophages. Overall, the total number of intercellular interactions in HCM decreased by approximately 50% (from 326 to 164), and the total interaction intensity dropped from 0.718 to 0.370, suggesting that myocardial hypertrophy significantly inhibits intercellular signal transduction (Figure 5A). The reduction in total interactions (from 326 to 164) was statistically significant (*p* < 0.001, permutation test). The decrease in total interaction intensity (from 0.718 to 0.370) was also significant (95% CI: 0.320–0.425 for HCM vs. 0.695–0.741 for non-failing, bootstrap). Communication between macrophages with fibroblasts, cardiomyocytes, and pericytes was notably weakened in HCM (Figure 5B). Signaling pathways, including PDGF, PCDH, FGF, MIF, and prostaglandin, were completely absent in HCM. The retained pathways were primarily structural ones, including COLLAGEN, LAMININ, and FN1, indicating a shift from regulatory signaling toward fibrosis-related signals in the HCM heart (Figure 5C). As shown in Figure 5D, the signal reception intensity of macrophages decreased from approximately 0.10 to about 0.03. This reduction indicates that in HCM, the ability of macrophages to receive signals from other cells is markedly impaired, reflecting compromised macrophage function.

### 2.6. Validation Expression of Target Genes in scRNA-Seq

**Figure 6:**
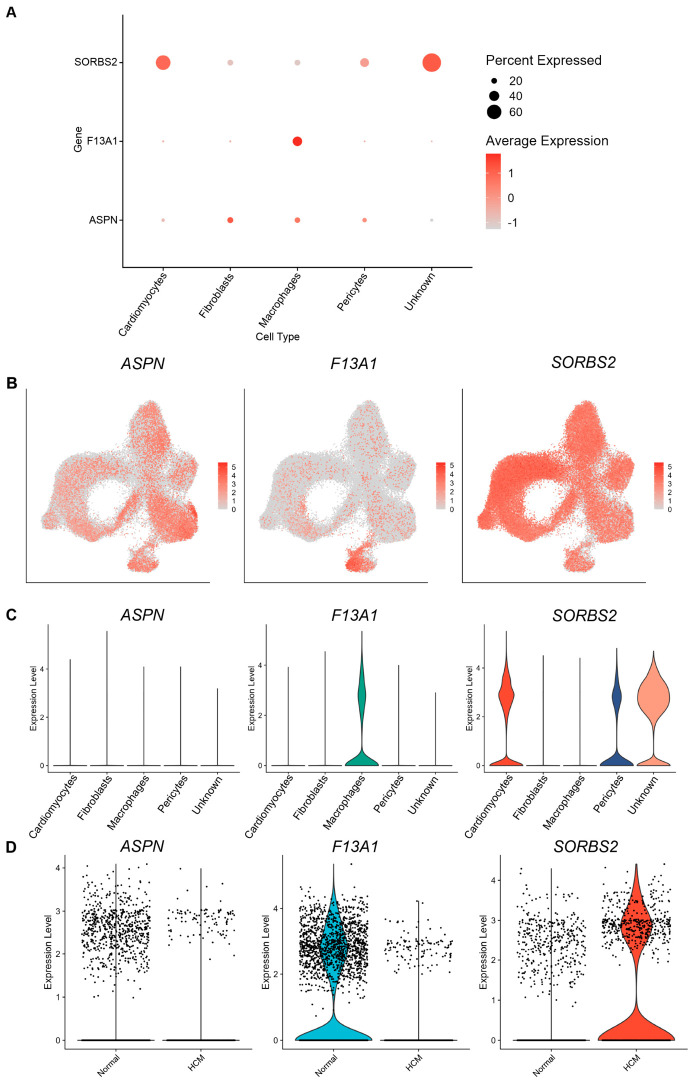
Expression profiling of target genes in scRNA-seq data. (**A**) Dot plot showing the expression of target genes (*ASPN*, *F13A1*, and *SORBS2*) across five cell types. (**B**) UMAP plots illustrating the expression distribution of target genes. (**C**) Violin plots displaying the expression levels of target genes in different cell types. (**D**) Expression levels of target genes specifically in macrophages in non-failing and HCM samples.

The above findings underscore the pivotal role of macrophages in HCM. To further validate the cell-type specificity of the three target genes (*ASPN*, *F13A1*, *SORBS2*) and compare their expression levels between HCM and non-failing samples, we analyzed the scRNA-seq data. As shown in Figure 6A, *F13A1* exhibited the strongest specificity for macrophages, with an average expression of 7.69 in macrophages compared to less than 0.33 in other cell types, confirming its specificity as a macrophage-related target gene. UMAP and violin plots revealed that *F13A1* expression was predominantly restricted to macrophage clusters. *SORBS2* was broadly expressed in cardiomyocyte and pericyte clusters, and *ASPN* showed generally low expression across all cell types (Figure 6B,C). We next compared the expression of these target genes specifically within the macrophage population between HCM and non-failing groups. *F13A1* was markedly downregulated in HCM (from 10.03 to 1.91), whereas *SORBS2* was significantly upregulated (from 1.64 to 7.47). These changes were consistent with the differential expression trends observed in the bulk RNA-seq data, thereby mutually validating the results across both platforms (Figure 6D).

### 2.7. Construction and Validation of the Machine Learning Diagnostic Model

**Figure 7:**
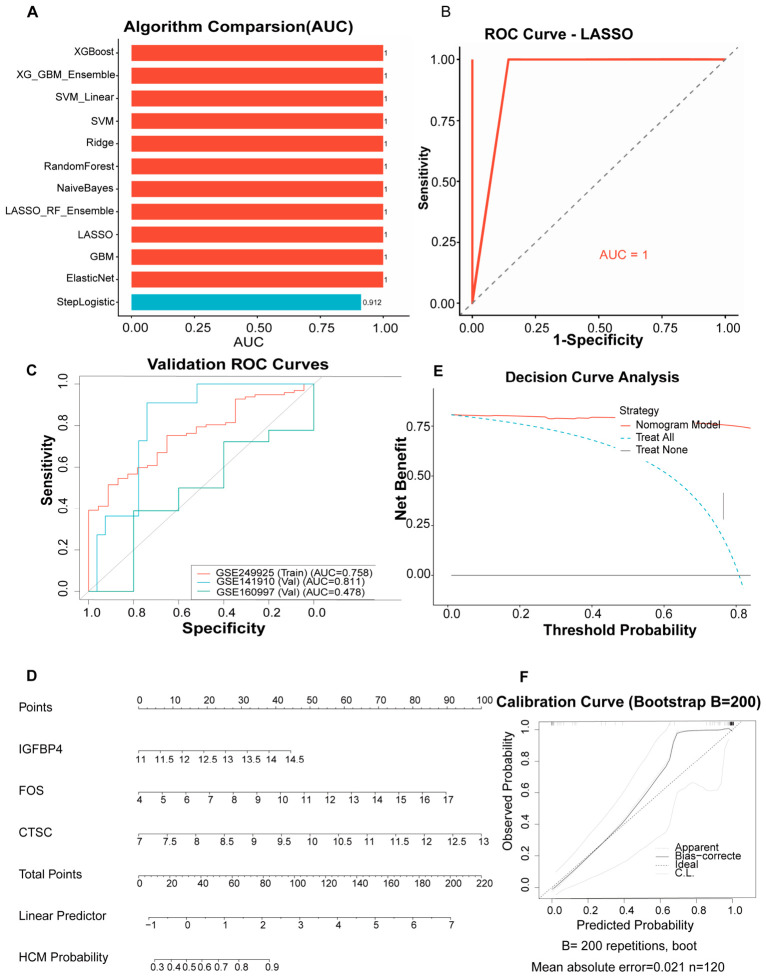
Identification and validation of signature genes and construction of the diagnostic model. (**A**) Comparison of area under the curve (AUC) values among 12 machine learning algorithms. (**B**) ROC curve of the LASSO model in the training set (GSE249925). (**C**) ROC curve of the LASSO model in the independent validation set (GSE141910). (**D**) Nomogram for estimating the total risk score for developing HCM. (**E**) DCA curves for the training. (**F**) DCA for the external validation cohort.

Based on 86 genes, a diagnostic model for HCM was constructed using 12 machine learning algorithms, and the optimal algorithm was selected through cross-validation. As shown in Figure 7A, the LASSO model achieved feature sparsity while maintaining good generalization ability, making it the optimal choice. Receiver operating characteristic (ROC) analysis performed on both the training cohort (GSE249925) and the external validation cohort (GSE141910) demonstrated that the three diagnostic genes (*IGFBP4*, *FOS*, *CTSC*) possess high predictive value and are closely associated with macrophages (Figure 7B,C). We further constructed a nomogram that quantifies the contribution of each feature variable, with the cumulative score representing the overall risk of developing HCM (Figure 7D). The decision curve analysis (DCA) showed that using the model to predict HCM risk provided more benefit than either the “all-treatment strategy” or the “no-treatment strategy” over a wide range of threshold probabilities, and demonstrated a favorable net benefit across different risk thresholds for both the training and validation cohorts, indicating that the model has certain clinical applicability (Figure 7E). In addition, the calibration curve exhibited high consistency between the predicted probability and the actual observed probability, with a C-statistic of 0.983 and a mean absolute error (MAE) of 0.021, suggesting excellent calibration performance of the model (Figure 7F). Collectively, these three macrophage-related genes exhibit strong diagnostic value and may serve as promising targets for clinical application.

## 3. Discussion

This study investigated transcriptomic differences between HCM patients and non-failing controls to elucidate the molecular mechanisms underlying the disease. Our integrative analysis has uncovered three macrophage-associated genes (*ASPN*, *F13A1*, and *SORBS2*) in HCM, with *F13A1* showing selective downregulation within the macrophage population. The pronounced impairment of intercellular communication, coupled with the absence of several regulatory signaling pathways in HCM hearts, points to a compromised macrophage-mediated immune response. Additionally, a LASSO-derived diagnostic signature based on *IGFBP4*, *FOS*, and *CTSC* demonstrated strong predictive accuracy, underscoring its promise as a clinical tool for HCM detection.

Validating the expression of target genes at the single-cell level, particularly confirming the specific high expression of *F13A1* in macrophages and its marked downregulation in HCM, substantially enhances its credibility as a biomarker. *F13A1* encodes the A chain of coagulation factor XIII, traditionally recognized for its role in the cross-linking and stabilization of fibrin during the terminal phase of coagulation. This gene has been implicated in various pathological conditions, including obesity [12], rheumatoid arthritis [13], tumor [14], and atherosclerosis [15]. Its high expression in tissue-resident macrophages suggests non-canonical functions, such as modulating extracellular matrix remodeling, inflammatory responses, and potentially influencing the tissue repair microenvironment [16]. Its downregulation may directly compromise the structural integrity of the extracellular matrix or indirectly impair macrophage-mediated inflammation resolution, thereby promoting the fibrotic pathology of HCM. In contrast, the upregulation of *SORBS2* in HCM macrophages is an intriguing observation; this gene encodes a protein involved in cytoskeletal organization and focal adhesion formation, and its upregulation may be associated with altered mechanosensing and migratory capacity of macrophages adapting to the pathologically stiffened matrix [17]. Regarding *ASPN*, although its expression level is low across all cell types, its encoded protein asporin is an important component of the extracellular matrix and regulates TGF-β activity [18]. Thus, subtle changes in its expression may produce amplified effects through modulation of key pro-fibrotic pathways, explaining why it was selected as a critical diagnostic gene despite its low expression abundance.

Notably, this study revealed a marked decrease in the proportion of macrophages in HCM cardiac tissues, a finding that contrasts with previous immune infiltration patterns inferred from bulk transcriptomic data [19]. Specifically, earlier studies using the CIBERSORT algorithm suggested higher abundances of macrophages (M0, M1, and M2 subtypes) in HCM samples compared with non-failing tissues [19], whereas our single-cell resolution direct counting revealed the opposite trend. This discrepancy likely arises from methodological limitations of computational approaches, as deconvolution-based algorithms may introduce bias when estimating cell proportions in complex tissues, thereby highlighting the advantage of direct single-cell sequencing for precise delineation of cellular composition. Further potential mechanisms underlying the reduced macrophage proportion include increased apoptosis, decreased recruitment from circulating monocytes, or phenotypic switching toward other cell types such as fibroblasts. A recent study in an HCM model identified an ENPP2-high, pro-inflammatory macrophage subset that activates fibroblasts through intercellular interactions, suggesting that macrophages may undergo functional reprogramming rather than merely a numerical reduction [1]. A complementary mechanistic study demonstrated that the activated fatty acid synthesis pathway in cardiac macrophages-via ACLY-mediated acetylation at the Krt17 promoter-drives the production of pro-fibrotic cytokines such as IL-33, thereby promoting the expansion of a pathogenic fibroblast subset highly expressing extracellular matrix genes after myocardial infarction [20]. This study also found that chronic inflammation downregulates SerpinB2 expression in tissue resident macrophages through the IFNγ pathway, leading to increased mitochondrial ROS, cytochrome c release, and macrophage apoptosis [21]. This results in increased insulin resistance and metabolic dysfunction—mechanisms that could also explain the reduced macrophage survival and disturbed immune homeostasis observed in patients with HCM. Moreover, another multi-omics study indicated that downregulation of endothelial-derived CX3CL1 may impair macrophage efferocytosis, representing a potential molecular pathway contributing to macrophage dysfunction and subsequent reduced clearance [5]. Beyond this, recent work has shown that RBPJ epigenetically controls efferocytosis by suppressing the repressive H3K9me3 mark on promoters of Stard13 and Arsg, thereby enhancing apoptotic cell clearance in macrophages [22]. Collectively, these findings suggest that both chemokine-mediated and epigenetic mechanisms may converge to regulate macrophage efferocytosis in HCM, though direct evidence in this disease context remains to be established.

From the functional enrichment analysis, differentially expressed genes in HCM were significantly enriched in complement and coagulation cascades, as well as MAPK and TNF signaling pathways, confirming from a systems biology perspective the intricate intertwining of immune inflammation with fibrotic progression. This finding corroborates multiple studies; for instance, in Fabry disease-associated HCM, activation of the NF-κB pathway driven by inflammatory cytokines has been linked to myocardial hypertrophic remodeling [23]. Of particular interest is the enrichment of the complement and coagulation cascade pathway, which may not only relate to classical inflammatory amplification but also to myocardial microvascular pathology and local microthrombus formation in HCM critical pathological link leading to myocardial ischemia and fibrosis. Regarding the MAPK and TNF signaling pathways, they constitute a central bridge connecting immune cell activation and parenchymal cell responses. For example, NLRC5 in macrophages regulates the secretion of cytokines such as IL-6 by inhibiting the NF-κB pathway, thereby influencing cardiomyocyte hypertrophy and fibroblast activation [8]. The pathway results of this study suggest that macrophage dysfunction may persistently activate MAPK and other signals within cardiomyocytes and fibroblasts through the release of specific cytokines, driving pathological remodeling. This aligns with another study showing widespread activation of AP-1 transcription factors (e.g., *FOS*, *JUN*) in cardiomyocytes in early-stage HCM models [8], indicating coordinated signaling network activation across different cell types.

Using the CellChat tool, this study revealed a global attenuation of the intercellular communication network in HCM hearts, particularly a marked decline in the signal-receiving capacity of macrophages, providing a novel perspective for understanding the underlying pathological mechanisms. This finding is broadly consistent with the overall trend of altered cellular communication reported in several recent single-cell studies on HCM, yet it unveils a more specific pattern of deficiency. For instance, one study noted reduced expression of ligand–receptor pairs involved in extracellular matrix and growth factor binding in HCM [24]; our study further quantified the decreased total number and strength of interactions and clearly identified the loss of regulatory signaling pathways such as PDGF and FGF, alongside a shift toward a pro-fibrotic COLLAGEN signaling predominance. The diminished communication capacity of macrophages, especially their impaired signal-receiving ability, may represent a key juncture in pathological remodeling rather than a mere consequence. Evidence indicates that the macrophage-specific molecule NLRC5, through interactions with other proteins, modulates macrophage functional status, thereby influencing the overall balance between inflammation and repair in the heart [8]. The elevated levels of γδ T cells and NK cells observed in this study may form a feedback loop with macrophage functional suppression, as certain immune cell subsets can affect macrophage polarization and communication capacity through cytokine secretion. Compared with dilated cardiomyopathy or myocarditis, this communication disorder pattern centered on macrophages, accompanied by enhanced pro-fibrotic signaling, may be more specific to HCM, consistent with findings from other studies comparing cellular communication signatures across different cardiomyopathy subtypes [25].

The core genes of the machine learning-based HCM diagnostic model (*IGFBP4*, *FOS*, *CTSC*) are not entirely consistent with the macrophage-associated genes identified through prior differential expression screening (*ASPN*, *F13A1*, *SORBS2*). This discrepancy precisely reflects the distinct objectives of diagnostic model construction versus mechanistic exploration. *ASPN*, *F13A1*, and *SORBS2* were identified through a stepwise multivariate regression aimed at selecting genes with independent diagnostic contribution for the purpose of mechanistic interpretation. These genes represent macrophage associated candidates that may directly participate in HCM pathogenesis. *IGFBP4*, *FOS*, *CTSC* were selected by the LASSO algorithm from the same pool of 86 macrophage related genes, but LASSO prioritizes predictive accuracy and parsimony through shrinkage, often choosing different features when multicollinearity exists. These three genes are not necessarily the most differentially expressed; rather, they form the most discriminative combination for distinguishing HCM from controls, making them suitable for a diagnostic model. Diagnostic models aim to select, through algorithmic approaches, the most informative combination of genes for discriminating between disease and control samples; these genes may serve as downstream or upstream hub nodes within disease-related pathways, rather than necessarily being the most significantly differentially expressed genes. For instance, *FOS*, a member of the AP-1 transcription factor complex, plays a central role in stress and growth factor signaling. Its activation in HCM cardiomyocytes has been documented in this study as well as in other work [26], and its expression changes may integrate inflammatory and hypertrophic signals originating from multiple cell types, including macrophages, thereby conferring strong diagnostic discriminatory power. *IGFBP4* is specifically cleaved by the protease PAPP-A, and elevated levels of its fragments correlate with cardiovascular complications and increased mortality in coronary artery disease, acute coronary syndrome, and heart failure [27]. Combining such model-derived genes with existing clinical parameters (e.g., echocardiographic measures) holds promise for constructing a multi-dimensional diagnostic system that improves the detection of early-stage disease or atypical cases, analogous to recent explorations of machine learning for differentiating HCM from cardiac amyloidosis and other conditions [28]. The three-gene signature (*IGFBP4/FOS/CTSC*) derived from macrophage-associated genes may support early detection and risk stratification of HCM. Future translational steps include: (1) prospective validation in independent multicenter clinical cohorts; (2) development of blood-based PCR or protein assays for noninvasive testing; (3) regulatory evaluation for clinical applicability. This study remains discovery-oriented; the identified genes are candidate biomarkers requiring further prospective verification before clinical use.

Based on single-cell and batch transcriptome data and CellChat analysis, the findings regarding macrophage dysfunction, impaired signal reception, and their pro-fibrotic effects are correlation rather than direct causal evidence. Previous studies have confirmed that the dysfunction of macrophage signal reception can directly affect its survival and activation, thereby promoting the proliferation and collagen deposition of fibroblasts [1]. The reduced number or functional defect of macrophages will lead to persistent inflammation and insufficient clearance of apoptotic cells, and eventually aggravate myocardial fibrosis [5,29]. Reduced macrophage–fibroblast communication in HCM is strongly associated with an imbalance in profibrotic pathways [7]. The results of this study are consistent with the conclusions of the above mechanistic studies, and provide a testable hypothesis for further exploration of the role of macrophages in the pathogenesis of HCM.

Several limitations should be acknowledged. First, all findings are derived solely from computational analyses of public datasets without experimental confirmation via qRT-PCR, Western blot, immunohistochemistry, or functional assays in independent patient cohorts or animal models; therefore, the reliability of the identified genes (*ASPN*, *F13A1*, *SORBS2*, *IGFBP4*, *FOS*, *CTSC*) as biomarkers or therapeutic targets for HCM remains to be determined by independent studies, and our results should be viewed as hypothesis-generating rather than conclusive. Large independent cohorts for subgroup analyses (age, sex, obstruction status) are currently unavailable. Further multicenter validation in stratified clinical subgroups is required to fully confirm the robustness of the identified genes and diagnostic model. Second, the scRNA-seq data include only 7 HCM and 6 non-failing hearts, which may not fully capture the heterogeneity of HCM across different genetic mutation subtypes or disease stages, and the number of macrophages in these data (4085 cells) is relatively modest, restricting our ability to perform fine-grained subclustering or to deeply explore the relationship between distinct macrophage functional states (e.g., M1/M2 polarization, efferocytosis) and HCM pathology. Moreover, the external validation set (GSE141910) is also small (only 11 HCM samples), which may lead to overestimation of the diagnostic model’s performance (AUC > 0.98), and multicenter, large-scale validation is needed before clinical translation. Finally, correlation does not imply causation: differential expression and cell–cell communication analyses reveal associations only, and they cannot distinguish whether macrophage dysfunction is a cause or a consequence of HCM. Despite these limitations, the integration of single-cell and bulk RNA-seq with rigorous machine learning provides biologically meaningful and clinically promising insights into macrophage-driven mechanisms in HCM.

## 4. Materials and Methods

### 4.1. Data Collection

All included cohorts consist of adult patients with clinically confirmed HCM and non-failing adult control hearts without known cardiomyopathy, heart failure, or significant coronary artery disease. No pediatric or end-stage heart failure patients were included. HCM diagnosis followed standard clinical criteria, including echocardiographic evidence of unexplained left ventricular hypertrophy and exclusion of other causes of hypertrophy. The scRNA-seq datasets used in this study were obtained from GSE181764 and GSE161921 comprising 7 HCM patients and 6 non-failing heart samples, which was utilized to characterize the cellular landscape in HCM cardiac tissue at single-cell resolution [30]. GSE249925 is a bulk RNA-seq dataset consisting of 23 non-failing individuals and 97 HCM samples, employed for differential expression analysis and machine learning model construction [31]. GSE141910 (27 non-failing individuals and 11 HCM) served as external validation sets to further evaluate the performance of the diagnostic model [32].

### 4.2. scRNA-Seq Data Processing

Quality control and preprocessing of the scRNA-seq data were performed using the Seurat package (version 5.0.1). Cells were filtered based on the following criteria: at least 200 detected genes, total gene counts >50, and mitochondrial gene content <25% to exclude low-quality and potentially dead cells. Doublets were identified and removed using the DoubletFinder package. After stringent quality control, high-quality cells were retained for downstream analysis. Batch effects across different samples were corrected using the Harmony package. Principal component analysis (PCA) was conducted for linear dimensionality reduction, followed by clustering and visualization. Cell clusters were identified using a graph-based clustering approach, and differentially expressed genes for each cluster were determined using the FindAllMarkers function, with filtering criteria set at average log_2_ fold change > 0.58 and expression in at least 25% of cells within the population. Cell types were annotated based on marker genes from CellMarker 2.0 and relevant literature. Low-quality cells and genes were filtered using the preprocess_cds function. Nonlinear dimensionality reduction was conducted using UMAP, and cells were clustered based on UMAP coordinates with the cluster_cells function. All scRNA-seq analyses were performed using R (v4.3.2) with Seurat v5.0.1, DoubletFinder, Harmony, and CellChat v1.6.1.

### 4.3. Bulk RNA-Seq Differential Expression Analysis

The bulk RNA-seq dataset GSE249925 was used to investigate the biological characteristics of HCM patients. Bulk RNA-seq dataset GSE249925 exclusively includes confirmed HCM patients; samples with dilated cardiomyopathy, restrictive cardiomyopathy, or heart failure of non-HCM etiology were excluded during data curation. Data processing included quality control, normalization, and differential expression analysis using the DESeq2 package (version 1.42.1). Low-expression genes-defined as those with counts <10 in fewer than 10% of samples-were filtered out. Normalization and differential analysis were performed using default parameters in DESeq2, with *p*-values adjusted by the Benjamini–Hochberg method. DEGs were identified using thresholds of |log2FC| > 1 and false discovery rate (FDR) < 0.05, and the results were visualized using volcano plots. Candidate genes were identified by intersecting DEGs derived from scRNA-seq and bulk RNA-seq datasets, and the overlap was visualized using a Venn diagram. To explore the potential biological functions of macrophage-related genes in HCM, GO and KEGG enrichment analyses were conducted using the ClusterProfiler R package (version 4.8.3). An adjusted *p*-value (p.adj) < 0.05 was considered statistically significant. Bulk RNA-seq analyses were performed using R (v4.3.2) with DESeq2 v1.42.1, clusterProfiler v4.8.3, pROC, glmnet, randomForest, xgboost, and gbm.

### 4.4. Immune Microenvironment Analysis

The enrichment scores of 19 immune cell subtypes were evaluated using ssGSEA. Briefly, the relative abundance of immune cells in each sample was estimated based on the expression of overall marker genes, with the enrichment score representing the relative proportion of each immune cell subtype. Differences in immune cell proportions between groups were assessed using the Wilcoxon rank-sum test. To further estimate immune infiltration levels, immune scores were calculated using the ESTIMATE R package. A *p*-value < 0.05 was considered statistically significant. Bubble plots were employed to illustrate the associations between key target genes and immune cells.

### 4.5. Cell Communication Analysis

To investigate intercellular communication networks in HCM, we performed cell–cell interaction analysis using the R package “CellChat” (version 1.6.1). The normalized gene expression matrix of scRNA-seq data from HCM and non-failing controls was used as input. For each cell type, we quantified the number and strength (interaction weight) of outgoing and incoming signals. The overall communication networks were compared between HCM and non-failing controls groups to identify differentially active signaling pathways. Pathway activity was ranked by the relative contribution of each signaling pathway to the total communication network. Changes in signal sending and reception intensities for each cell type were visualized using scatter plots. The robustness of the inferred interactions was assessed by a permutation test. This analysis allowed us to characterize how HCM alters intercellular signaling, with a particular focus on macrophages and their interactions with other cardiac cell types. To statistically assess the differences in total interaction number and total interaction strength between HCM and non-failing groups, we performed permutation testing (1000 permutations, shuffling group labels) to obtain a *p*-value for the interaction count difference, and bootstrap resampling (1000 iterations with replacement) to calculate 95% confidence intervals for the interaction intensity difference.

### 4.6. Machine Learning Diagnostic Model Construction and Evaluation

#### 4.6.1. Feature Selection

Candidate genes were derived from the intersection of scRNA-seq macrophage markers and bulk RNA-seq DEGs, yielding 87 genes. Univariate logistic regression (*p* < 0.05) further filtered these to 86 macrophage-related genes for model input, reducing noise and dimensionality.

#### 4.6.2. Dataset Partitioning

The GSE249925 cohort (*n* = 120) was randomly split into a 70% training set (*n* = 83) and a 30% internal test set (*n* = 37) using stratified sampling by disease status, with a fixed random seed (2026) for reproducibility. For hyperparameter tuning, we applied 10-fold cross-validation repeated 10 times exclusively on the training set, selecting the optimal lambda value via cv.glmnet for the LASSO model. Importantly, the external validation set GSE141910 (*n* = 38) was kept completely independent throughout feature selection, model training, and hyperparameter tuning, and was only used for final performance evaluation.

#### 4.6.3. Algorithms and Cross-Validation

A total of twelve machine learning algorithms were applied, including LASSO (Least Ab-solute Shrinkage and Selection Operator) regression, Ridge regression, Random Forest, Support Vector Machine (SVM), SVM_Linear, Gradient Boosting Machine (GBM), XGBoost (Extreme Gradient Boosting), NaiveBayes, Elastic Net, and Step_Logistic, along with ensemble models that integrated multiple algorithms. 10-fold cross-validation (10 repeats) was used to tune hyperparameters for LASSO, Ridge, and Elastic Net. Tree-based models (Random Forest, XGBoost, GBM) used 5-fold cross-validation with fixed empirical parameters, avoiding extensive grid search.

#### 4.6.4. Model Evaluation

Model performance was assessed using AUC. LASSO was selected as the optimal model due to its sparsity and generalizability. Final evaluation relied on external validation (AUC = 0.811) rather than internal results. Calibration curves (MAE = 0.021) and decision curve analysis further confirmed model reliability.

#### 4.6.5. Overfitting Prevention

Three strategies minimized overfitting: (1) Strict feature filtering to retain only biologically relevant genes; (2) L1 regularization (LASSO) to reduce model complexity; (3) Independent external validation as the primary performance metric. All analyses were performed in R with a fixed random seed (2026) for reproducibility.

### 4.7. Statistical Analysis

All statistical analyses were conducted using the R software (versions: R 4.3.2). DEGs between HCM and non-failing control samples were identified with|log2(FC)| > 1 and p.adj < 0.05. Enriched GO or KEGG items were identified by using the threshold of p.adj < 0.05. Spearman’s correlation analysis was utilized to explore the relationship between target gene and immune cell infiltration.

## 5. Conclusions

In summary, by integrating single-cell and bulk transcriptomics, this study presents the first systematic delineation of macrophage-mediated immune microenvironment dysregulation and remodeling of cellular communication networks in HCM. Moreover, we established a machine learning-based diagnostic model exhibiting considerable potential for clinical translation. These findings not only provide novel insights into the immunopathological mechanisms underlying HCM but also establish a theoretical foundation for developing early diagnostic tools targeting macrophage-associated genes. Future research should validate the diagnostic performance of this model in prospective clinical cohorts and employ functional experiments to elucidate the precise molecular mechanisms by which key target genes contribute to myocardial hypertrophy and fibrosis.

## Data Availability

The data is contained within the article or the Supplementary Materials.

## Supplementary Materials

The following supporting information can be downloaded at: https://www.mdpi.com/article/10.3390/ijms27115102/s1.

## Abbreviations

The following abbreviations are used in this manuscript:

ASPN	Asporin
AUC	Area under the curve
DCA	Decision curve analysis
DEG	Differentially expressed genes
F13A1	Factor XIII A Chain
FDR	False discovery rate
GBM	Gradient Boosting Machine
GO	Gene Ontology
HCM	Hypertrophic cardiomyopathy
KEGG	Kyoto Encyclopedia of Genes and Genomes
MAE	Mean absolute error
PCA	Principal component analysis
ROC	Receiver operating characteristic
scRNA-seq	Single-cell RNA sequencing
SORBS2	Sorbin and SH3 domain-containing protein 2
ssGSEA	Single-sample gene set enrichment analysis
SVM	Support Vector Machine
XGBoost	Extreme Gradient Boosting
