# Supplementary material for: Integrative Transcriptomics and Machine Learning Identify Macrophage-Associated Biomarkers in Hypertrophic Cardiomyopathy
Source: Int J Mol Sci. 2026 Jun 4;27(11):5102. doi: 10.3390/ijms27115102 (PMC13256712; doi:10.3390/ijms27115102)
Supplement: Supplementary file 1 [file ijms-27-05102-s001.zip › Supplementary figures.pdf]

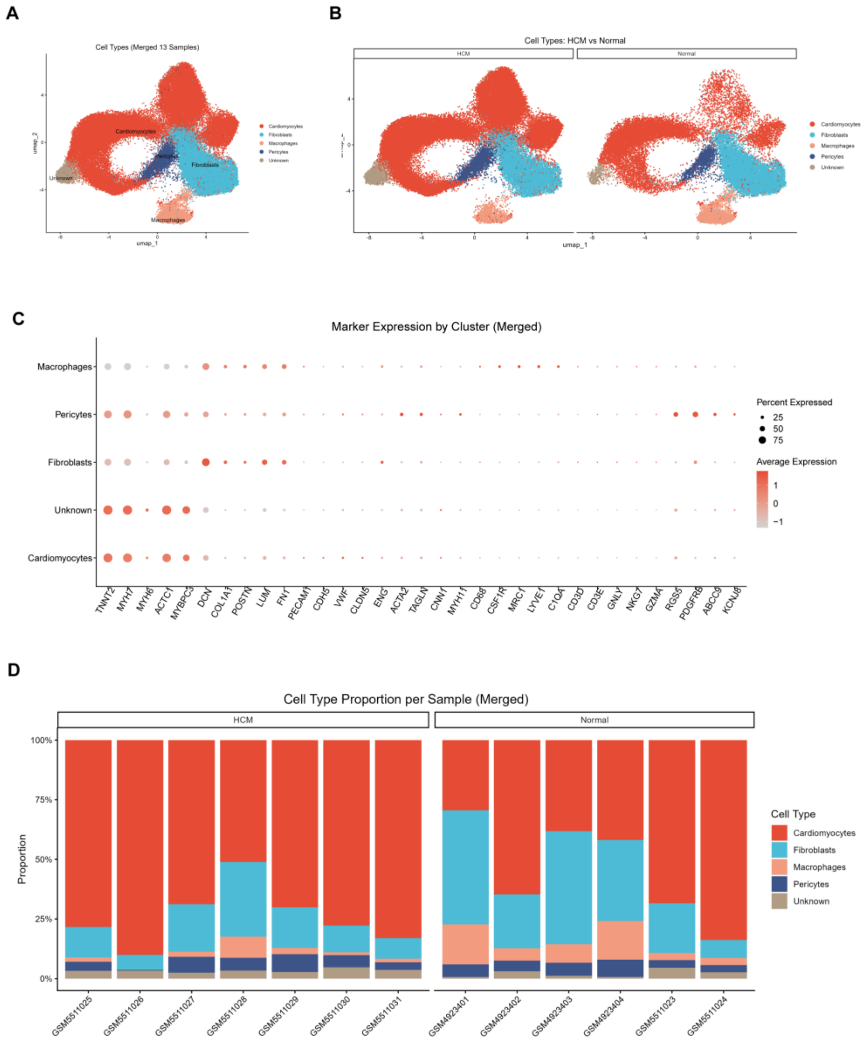

Figure S1. Single-cell RNA sequencing reveals major cell populations in cardiac tissues. (**A**) UMAP plot showing five major cardiac cell clusters, with each color representing a distinct cell type as indicated in the legend. (**B**) UMAP plot of annotated cell clusters in HCM and non-failing control groups. (**C**) Expression levels of canonical marker genes across the five identified cell types. (**D**) Relative proportions of each cell type in non-failing control and HCM samples.

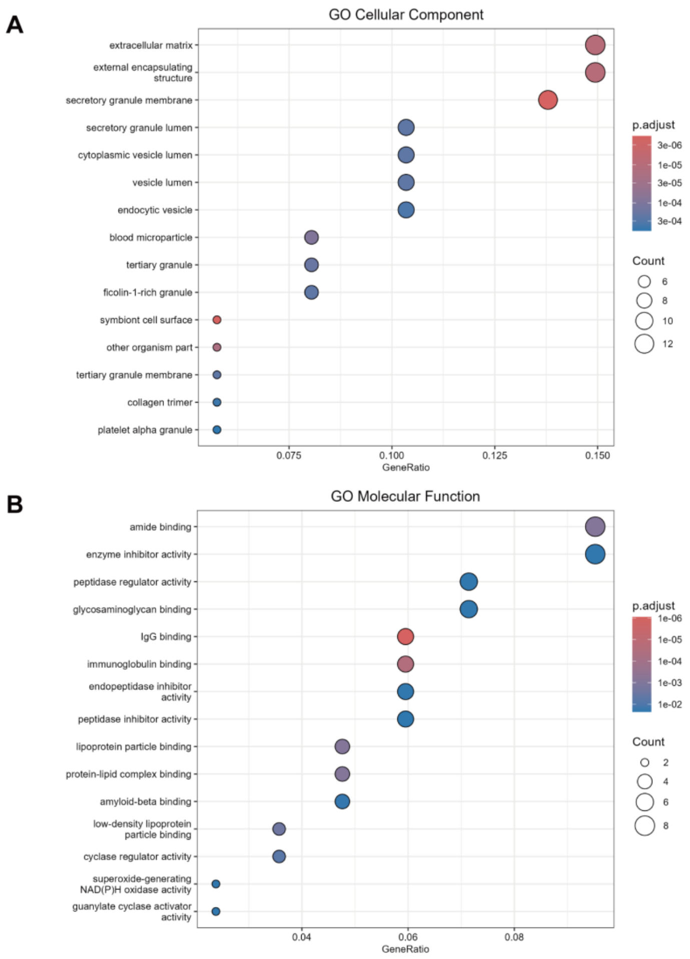

Figure S2. Gene Ontology enrichment analysis.
(**A**) Gene Ontology Cellular Component (CC) enrichment analysis. (**B**) Gene Ontology Molecular Function (MF) enrichment analysis.
